# The biology of chronic myeloid leukemia: an overview of the new insights and biomarkers

**DOI:** 10.3389/fonc.2025.1546813

**Published:** 2025-05-08

**Authors:** Anna Sicuranza, Alessia Cavalleri, Simona Bernardi

**Affiliations:** ^1^ Chair of Hematology, University of Siena, Azienda Ospedaliera Universitaria, Siena, Italy; ^2^ Chair of Hematology, Department of Clinical and Experimental Sciences, University of Brescia - Unit of Blood Disease and Stem Cell Transplantation, ASST-Spedali Civili, Brescia, Italy; ^3^ Laboratorio CREA (Centro di Ricerca Emato-oncologica AIL), ASST-Spedali Civili, Brescia, Italy; ^4^ National Center for Gene Therapy and Drugs based on RNA Technology (CN3), Padua, Italy

**Keywords:** CML, biological markers, BCR::ABL1, *ASXL1*, EVs, CD26, leukemic stem cell, miRNA

## Abstract

Chronic myeloid leukemia is one of the onco-hematologic diseases in which the identification of disease markers and therapeutic advances have been particularly impactful. Despite this, significant gaps remain in our understanding of disease pathogenesis, progression, mechanisms of immune escape, and resistance to standard therapies. Recently, advances in technology and biological knowledge have drawn attention to several promising areas of research. Among these, leukemic stem cells, miRNAs, extracellular vesicles, and additional BCR::ABL1 mutations, with particular reference to the *ASXL1* gene, have been the most extensively investigated. In this review we summarized and critically commented the main findings on these key topics over the past 5 years, evaluating their potential impact on patient management and their role in the development of new therapeutic strategies.

## Introduction to chronic myeloid leukemia

**Figure 1:**
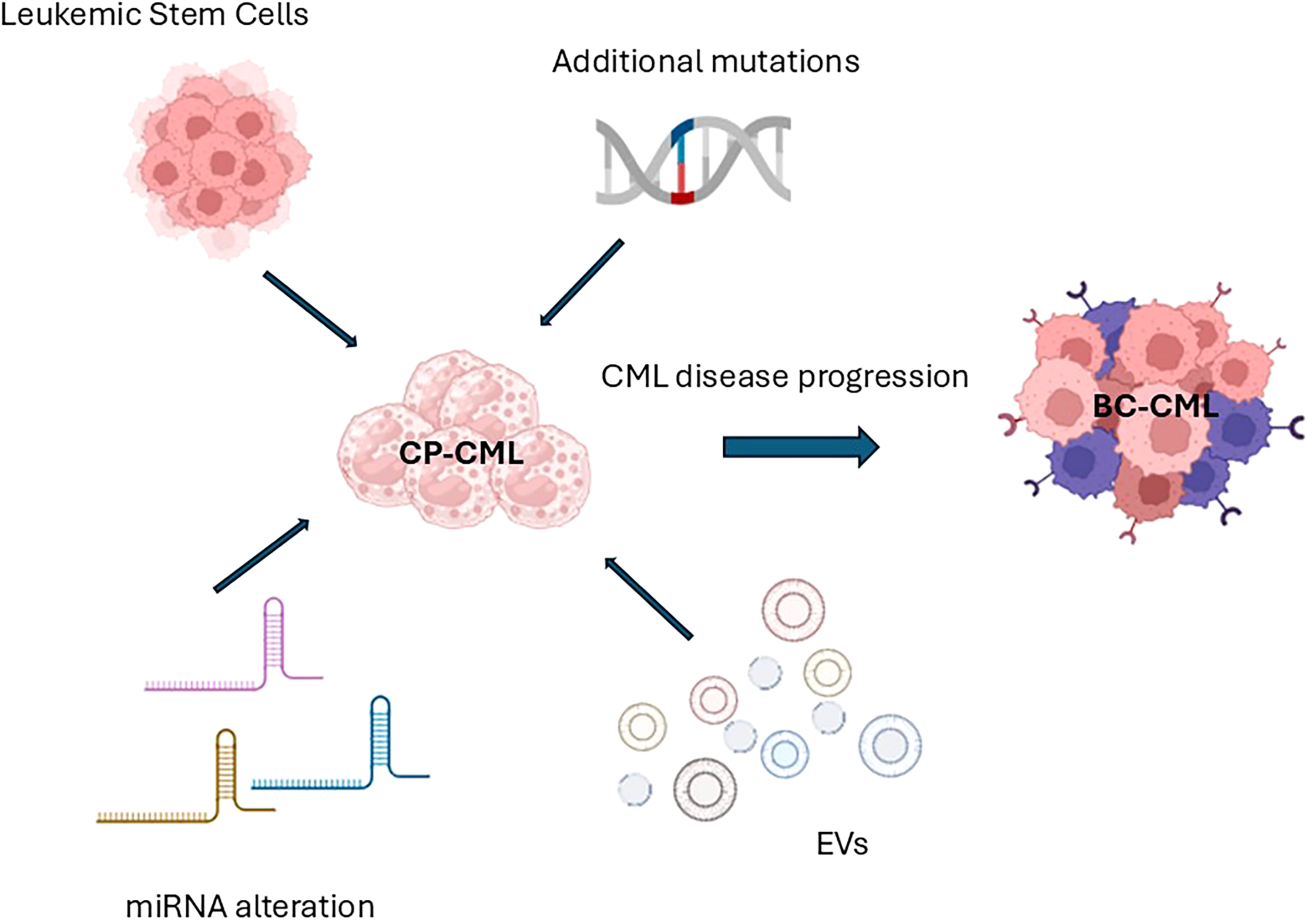
Schematic representation of the main biological characters investigated in the present review (Leukemic stem cells, mutations different from BCR::ABL1, miRNA, and EVs) and their influences on disease progression (miRNA, microRNA; EVs, Extracellular Vesicles; CP-CML, Chronic Phase Chronic Myeloid Leukemia; TKI, Tyrosine Kinase Inhibitor; BM, Bone Marrow; BC – CML, Blast Crisis or Blast Phase Chronic Myeloid Leukemia).

The uncontrolled proliferation of myeloid cells at various stages of maturity, found in both bone marrow (BM) and peripheral blood (PB), is the hallmark of chronic myeloid leukemia (CML), a hematologic malignancy. Traditionally classified into three phases, chronic (CP), accelerated (AP), and blastic phases (BP), CML has served as a pioneer in numerous therapeutic advancements (1). The Philadelphia (Ph)-chromosome, or translocation t (9,22), was identified as the cytogenetic hallmark of CML, and the *BCR::ABL1* fusion gene was later recognized as the central pathophysiological driver of the disease. The 210 KDa chimeric protein encoded by BCR::ABL1 exhibits constitutively active tyrosine kinase activity, stimulating multiple downstream signaling pathways in leukemic cells (2). Specifically, the expression of this oncoprotein alters cell adhesion to stromal components and the extracellular matrix, enhancing survival and inhibiting apoptosis (3). Furthermore, it promotes the acquisition of self-renewal capacity and cellular transformation. The cornerstone of modern CML therapy is the use of tyrosine kinase inhibitors (TKIs). Their introduction has resulted in high remission rates and significantly improved patient survival. This paradigm shift has moved the clinical focus from patient salvage to improved monitoring and quality of life (4–10). Among approved TKIs, imatinib, dasatinib, nilotinib, and bosutinib are recommended as frontline treatments for CML patients according to current guidelines (11, 12). Conversely, individuals who have received two or more TKI treatments in the past or who have the T315I mutation are the target of third-generation TKI ponatinib and the more recent asciminib (13, 14). Although CML therapy has achieved remarkable progress, the disease still poses significant clinical challenges due to its unpredictable progression and prognosis, as well as the highly individualistic nature of the CP’s duration and response to treatment. In fact, CML has recently become a subject of interest for Artificial Intelligence (AI)- based approaches, which aim to enhance prognostic accuracy and optimize prediction of treatment response (15–17). Indeed, it is well recognized that the BCR::ABL1 oncoprotein contributes to the acquisition of additional genetic lesions, likely as a result of increased genomic instability (18, 19). The consequences of this clonal evolution include a higher risk of relapse, poorer prognosis, resistance to TKI therapy, and, unfortunately, progression to BP-CML (20, 21). For these reasons, ongoing biological research aimed at further elucidating the molecular mechanisms of CML remains essential (22). In the present review, we focused on the study of Leukemic Stem Cells (LSCs), microRNA (miRNA), Extracellular Vesicles (EVs) and genomic mutations others than BCR::ABL1, with particular emphasis on *ASXL1* (**Figure 1**).

CD26+LSCs, which are specific to CML, have garnered considerable attention due to their persistence at diagnosis, during treatment, and even in treatment-free remission (23). They have been implicated in TKI resistance and disease relapse following therapy (24–26). In parallel, the expression of various miRNAs plays a role in the development, progression, and drug resistance of CML. Additionally, EVs have been investigated for their ability to modulate the microenvironment and immune system and transfer leukemic elements like BCR::ABL1 to recipient cells (27, 28). Finally, mutations in the *ASXL1* gene, particularly in genes involved in epigenetic regulation and DNA repair, have been linked to treatment resistance and rapid disease progression (29–31). These mutations are crucial in identifying high-risk patients who may need alternative or intensified treatment approaches (29, 32–35).

## Material and methods

We performed an electronic search in the PubMed database to find papers published between 2020 and 2025 and critically evaluated their potential implications for the improvement of the CML analysis and understanding. The search strategy included terms such as “CD26” (or DPP-4, or DPPIV), “Leukemia Stem Cells”, “miRNA”, “extracellular vesicles” (or EVs), “mutations” (or ASXL1) in combination with “CML” or “Chronic myeloid leukemia”. In this review, we summarized the information extracted from original studies with the aim of providing a comprehensive overview of the role of these biological markers and their potential clinical applications in the management of CML. Additionally, for each section of the review, we specified the number of articles found, those excluded for being off-topic or for other reasons, and the number of articles ultimately analyzed.

### Leukemic stem cells: lights and shadows

An important milestone in the timeline of CML research is represented by the study of Leukemia stem cells (LSCs). In recent years, several studies exploring the presence and persistence of LSCs have been conducted (36, 37). As is now known, CML-LSCs reside in the CD34+/CD38- Lin- cellular fraction and express the antigen CD26+ (dipeptidyl peptidase IV or DPP-4), which is considered the specific marker of the LSCs of CML. It is not expressed in the normal hematopoietic stem cells or in LSCs of other hematological diseases (38–41). Furthermore, retrospective flow cytometry studies have demonstrated that CD26+LSCs are detectable in PB samples in addition to BM aspirates, as previously documented (42). Moreover, it has been demonstrated that all CML patients at diagnosis express CD26+LSCs, with variable values, and that a fraction of these residual cells has been measured during TKI treatment and during TFR, without a clear correlation to molecular response (43). Following these preliminary observations, Bocchia and colleagues conducted prospective multicenter studies to better understand the behavior of these residual cells during TKIs treatment and during the TKI discontinuation. Prospective data showed that PB CD26+LSCs are measurable during TKIs treatment at the same time-points of molecular evaluations (at 3, 6, 12 and 24 months of TKI therapy), although their levels are drastically reduced compared to baseline values at diagnosis. Consequently, researchers investigated the role of the CD26+LSCs burden in PB at diagnosis in relation to treatment response. Data obtained from the “Prospective Flowers Study” suggested for the first time the correlation between the bulk of CD26+LSCs at diagnosis and the response to TKI treatment. Specifically, a lower number of CD26+LSCs at diagnosis was associated with an optimal molecular response at 3, 12, and 24 months (*BCR::ABL1* <10% and <0.1%, respectively) (44). In detail, in CML patients with optimal molecular responses at 3 months (*BCR::ABL1* <10%), the median CD26+LSCs at diagnosis was 6,21 cells/µl, compared to 19,87 cells/µl of suboptimal responders. In patients with an optimal molecular response at 12 and 24 months (*BCR::ABL1* <0.1%), the median CD26+LSCs counts at diagnosis were 5,50 cells/µl and 6,05 cells/µl respectively, compared to 16,87 cells/µl and 20,52 cells/µl of suboptimal responders (44). Instead, at the time of TKI withdrawal, approximately half of CML patients showed detectable but lower level of PB CD26+LSCs, regardless of their prior TKI treatment. No significant correlation was observed between the persistence of PB CD26+LSCs and the relapse rates (45).

Although the CD26+LSCs expression has been fully investigated in these last years, confirming their involvement in TKI resistance and disease progression, the mechanisms supporting LSCs survival remain unclear, and several questions are still unanswered. How do these leukemic cells remain quiescent? Are they able to elude the immune system? If so, by what strategies? Are there other features of these cells that are not yet known?

We conducted an electronic search on PubMed to examine the presence of recent publications addressing these unresolved issues. We obtained 81 results in the timeframe from 2020 to 2025. Several articles were rejected for being off-topic. After a careful selection process, we included only a few scientific articles considered novel and original. Two noteworthy recent research papers (published in December 2024 and January 2025) investigated the cellular heterogeneity of CML by using single-cell multiomics and single-cell proteo-transcriptomic analysis. In the first study, data reveal that antigens CD26 and CD35 play distinct roles in CML, and their interaction helps to differentiate between LSCs and normal hematopoietic stem cells (HSCs). CD26+CD35- cells are identified as leukemic and express high levels of BCR::ABL1, while CD26-CD35+ cells are healthy and have low or negative levels of BCR::ABL1. This distinction is crucial for understanding CML cellular heterogeneity and for developing targeted and effective therapies (46). The second paper investigated the effects of hydroxyurea (HU) treatment on stem and progenitor cells (SPC) in early-phase CML, suggesting that initial HU treatment modifies the characteristics of LSCs in CML and that studies on LSC and progenitor populations in CML should take into account the effects of HU therapy (47). Instead, another study demonstrated the existence of a distinct cell phenotype, in a subgroup of CD34+ stem cells, known as very small embryonic-like stem cells (VSELs) that are less sensitive to apoptosis than leukemic hematopoietic stem cells (LHSCs) after imatinib treatment. The study revealed that imatinib induces apoptosis and reduces proliferation and Ki67 mRNA expression more effectively in LHSCs than LVSELs. Moreover, some miRNAs, such as miR-451, miR-126, and miR-21, showed a significant increase in their expression in LVSELs compared to LHSCs after imatinib therapy. Nevertheless, further loss- or gain-of-function experiments in cell line models are necessary to validate these findings (48). Wang and colleagues explored the mechanism of survival of LSCs demonstrating their high expression of the hypoxia-inducible factor 2a (HIF-2a), which is responsible for controlling the metabolic state of the niche (49). Regarding the survival ability, the role of YBX1 has been investigated on CML cell lines and mouse models. The YBX1 expression is high in CML cells confirming its ability in regulation of the expression of gene related to the apoptosis (50). Dr. Scott conducted a study to demonstrate that the regulatory programs of quiescent LSC in CP-CML are analogous to that of embryonic stem cells; he displayed the role of wild type p53 in LSC self-renewal. Experiments on cell lines and CML double transgenic (DTG) mouse models showed that a p53 activation by the MDM2 inhibition could be considered an optimal strategy to inactivate quiescent LSC in the presence of TKI (51).

Regarding the role of the LSC niche in drug resistance, we selected two interesting studies conducted by Valent P. and Shi R, respectively. In the first, the authors demonstrated that osteoblasts are a major site of niche-mediated LSC resistance against TKI and identified PI3 kinase and mTOR as drugs capable of suppressing the growth and viability of osteoblasts and/or other niche cells, suggesting their use to neutralize the drug resistance of LSCs (52). The second study, on the other hand, explored the role of glycoprotein CD44 in microenvironmental communication, involving cell survival, resistance and persistence (53). An Argentinian study analyzed by NGS, the miRNome of the cellular fraction CD34+CD38-CD26+ vs. HSC obtained from the same CP-CML patients, comparing with stem cells from healthy donors. Levels of miR-196a-5p were significantly higher in CD26+ compared to the CD26- fraction at diagnosis, and in silico analysis showed an association with lipid metabolism and haematopoiesis functions, suggesting the relevant role of miRNAs in the cellular metabolism (54).

These data add novel interesting information to the study of LSCs, confirming their heterogeneity and association with response to treatment. However, several questions remain unanswered, and additional studies are needed to fully elucidate the involvement of LSCs in the TKI resistance. One hypothesis could be the ability of these cells to ‘survive’ the immune system, which would partly explain why CD26+ cells remain in circulation even many years after patients have discontinued TKIs, resulting in disease relapse. Although, at this moment, no data confirming these suggestions are available. It remains to be investigated and understood how these cells escape or elude the immune system. What mechanisms are activated? Are other molecules involved?

### Dysregulation of microRNA in CML: role in progression of disease and drug resistance

miRNA are short and non-coding RNAs consisting of 20–25 nucleotides that bind the 3’untranslated (3’UTR) region of specific mRNA target by regulating the gene expression at the post-transcriptional level (55). In addition to their canonical role, miRNAs can also function through non-canonical mechanisms such as binding to non-3’ UTR regions, miRNA-mediated activation, miRNA sponging, regulation of transcription, and epigenetic modulation (56). The miRNA origin consists of a series of processes foreseeing an initial conversion of primary miRNA (pri-miRNA) to a miRNA precursor (pre-miRNA) by the Drosha RNAse III enzyme in the nucleus. Subsequently, the pre-miRNA is exported from nucleus to cytoplasm by exportin-5 protein and cleaved by another RNAse III enzyme (Dicer) in the functional mature miRNA (57). miRNAs are present in body fluids such as blood, serum and plasma (58).

The first miRNA was identified in 1993 in a clone of C. elegans (59) and afterward numerous miRNAs have been discovered in cellular mechanisms, confirming their important role. Several studies demonstrated that miRNAs are involved in human cancers, including hematological disease. Particularly, primary studies on cancer cells demonstrated a reduced expression of mature miRNA, which should be associated with an upregulation of the mRNA target, leading to aberrant gene expression (60).

Although over the years, several miRNAs associated with CML and therefore with the BCR::ABL1 expression have been identified, in this review we summarized the most recently studied miRNA and focused on different roles in development, progression and drug resistance, exploring their biological significance and potential clinical impact.

We performed an electronic search to find papers in the PubMed database, considering only the time frame 2020–2025 years.

From the search a total of 133 publications have been obtained; 47 of them were off-topic and discarded. Editorials or case reports were excluded. Publications describing the same miRNAs were compared and merged. Of the remaining 86 publications, 21 manuscripts have been selected, based on the type (review article, systematic article or original article) and free full access.

Due to their great variety, miRNAs can be divided into two large categories based on their association with different biological states or responses to TKIs treatment.

miRNAs associated with cell proliferation and apoptosis:

- miR-7-5p, miR-17-92, miR-21, miR-29a-3p, miR-152-3p, miR-155-5p, miR-181, miR-221, miR-362-5p, miR-486-3p, miR-486-5p: overexpression of these miRNAs in CD34+ CML cells causes their proliferation, inhibition of phases G0/G1 of the cellular cycle, and induction of apoptosis (61–66);
- miR-10a: functions as a tumor suppressor by regulating the expression of upstream stimulatory factor 2 (USF2) in CML. Reduced levels of miR-10a-5p are observed in CD34+ cells from CML patients (67);
- miR-15a-5p: negatively regulates cell survival and metastasis by targeting CXCL10 in CML (68);
- miR-30a: functions as an autophagy inhibitor by downregulating the expression of autophagy proteins ATG5 and Beclin-1. miR-30a mimic amplifies the cytotoxicity generated by imatinib and promotes apoptosis (61);
- miR-188-5p: acts as an oncomiRNA in CML pathogenesis by upregulating BUB3 and SUMO2, promoting cell proliferation and inhibiting apoptosis (69);
- miR-21, miR-23a, miR-29b, miR-122, miR-126, miR-138, miR-140-5p, miR-142a-3p, miR-142a-5p, miR-146a, miR-150, miR-153-3p, miR-181c, miR-196b, miR-199a-3p, miR-203, miR-217, miR-223, miR-320a, miR-326, miR-342-5p, miR-370-3p, miR-379-5p, miR-409-5p, miR-424-5p, miR-451, miR-570-3p: are responsible for cell proliferation and promoting apoptosis. Several of these miRNAs play a role in regulating hematopoiesis or in immune response and act as tumor suppressors by targeting genes involved in cell proliferation (66, 70–72).

miRNAs Influenced by Tyrosine Kinase Inhibitors (TKIs):

- miR-17-92: reduced in response to imatinib treatment (65, 66);
- miR-181a: levels are elevated in imatinib-resistant cells; inhibiting miR-181a may enhance the effectiveness of imatinib and other TKIs (65, 66, 71);
- miR-342-5p: reduced expression of miR-342-5p contributes to CML progression and imatinib resistance. Enhancing miR-342-5p expression may improve response to imatinib (65, 73);
- miR-21: reduced expression after 6 months of imatinib (61, 74);
- miR-146a, miR-122, miR-126: higher expressed in responder patients compared with non-responders after 3 and 6 months of imatinib treatment, respectively (61, 75, 76);
- miR-106a and miR-155: higher expressed in patients resistant to TKI treatment and associated with higher bone marrow microvessel density (MVD) (77);
- miR-203: higher in imatinib responder patients and lower in non-responders; down-regulated in CML cells, miR-203 suppression leads to increased BCR::ABL1 activity. Restoring miR-203 levels could help suppress BCR::ABL1 and induce apoptosis (78).

**Table 1** summarizes the main dysregulated miRNA in CML.

**Table 1:** Dysregulated miRNAs in CML.

miRNA Associated with Cell Proliferation and Apoptosis	Function	Ref.
miR-7-5p, miR-17-92, miR-21, miR-29a-3p, miR-152-3p, miR-155-5p, miR-181, miR-221, miR-362-5p, miR-486-3p, miR-486-5p	Promote cell proliferation and apoptosis	(61–66)
miR-10a	Tumor suppressor	(67)
miR-15a-5p	Regulates cell survival and metastasis	(68)
miR-30a	Inhibits autophagy, promotes apoptosis	(61)
miR-188-5p	Promotes cell proliferation, inhibits apoptosis	(69)
miR-21, miR-23a, miR-29b, miR-122, miR-126, miR-138, miR-140-5p, miR-142a-3p, miR-142a-5p, miR-146a, miR-150, miR-153-3p, miR-181c, miR-196b, miR-199a-3p, miR-203, miR-217, miR-223, miR-320a, miR-326, miR-342-5p, miR-370-3p, miR-379-5p, miR-409-5p, miR-424-5p, miR-451, miR-570-3p	Regulate hematopoiesis, immune response, act as tumor suppressors	(60, 66– 70)
miRNAs Influenced by Tyrosine Kinase Inhibitors (TKIs)		
miR-17-92	Reduced by imatinib	(65, 66)
miR-181a	Elevated in imatinib-resistant cells	(65, 66, 71, 79)
miR-342-5p	Reduced expression contributes to CML progression	(65, 73)
miR-21	Reduced after 6 months of imatinib	(61, 74)
miR-146a, miR-122, miR-126	Higher in responders to imatinib	(61, 75, 76)
miR-106a, miR-155	Higher in TKI-resistant patients	(77)
miR-203	Higher in imatinib responders, lower in non-responders	(78)

Observations from these studies revealed the importance of investigating the miRNA and particularly studying the correlation between their expression and the status of the disease, to better understand the potential significance of these molecules on the development of the disease, but also their possible role as therapeutic targets.

Recent studies promoted miRNA-based therapeutic approaches, such as synthetic miRNA mimics or anti-miRNA Oligonucleotides (AMOs). The first are artificially created molecules replicating the function of naturally occurring miRNAs. They can be used to restore the function of down-regulated miRNAs, such as miR-203 and miR-342-5p, thereby suppressing oncogenic pathways and promoting apoptosis (78, 80, 81). AMOs are short, synthetic strands of nucleotides designed to bind to specific miRNAs and inhibit the function of miRNAs that are up-regulated such as miR-155 and miR-181a, reducing their oncogenic effects and enhancing the efficacy of existing therapies (79, 80). Therefore, in light of these evidences, the study of dysregulation of miRNAs plays a significant role in CML and needs to be fully investigated to add furthers information for developing new tools for the management of CML and the improvement of patient outcomes.

### Extracellular vesicles: what they shuttle and which are the effects

Both eukaryotic and prokaryotic cells secrete extracellular vesicles (EVs), a wide variety of cell-derived membrane structures that include proteins, lipids, and nucleic acids. EVs have been identified in several biological fluids and are found to be secreted by the majority of cell types. Based on their physical traits and biogenesis mechanism, EVs can be broadly divided into two subtypes: small-EVs and large-EVs. As previously indicated, it is now well documented that EVs play a crucial role in mediating intercellular communication and can transmit bioactive chemicals from their original cell to another in both healthy and pathological pathways (82). In fact, released EVs may interact with various cell types both near and distant from the cell of origin, serving as autocrine mediators for the releasing cells. They do indeed function as endocrine or paracrine mediators. By transporting a variety of substances, including proteins, lipids, and the previously mentioned nucleic acids, many of which are carefully sorted inside the vesicles, EVs facilitate information flow between cells (28, 83). It can be stated that phenotypic changes in the cell of origin are reflected in the composition of the secreted EVs, both in terms of EV type and cargoes, as it is evident that the composition of EVs is mainly dependent on the status of the producer cells. Hematopoietic and mesenchymal stem cells, myeloid-derived suppressor cells, and endothelial cells (ECs) are the primary cells with which the leukemic cell communicates in the context of CML through sEVs (84, 85). Tumor-derived EVs are known to have an extensive impact on the various receiving cells. In fact, they continue to play a vital role as mediators in important cancer processes due to their effects on cellular proliferation and resistance to apoptosis, promotion of angiogenesis, transfer of mutations, and modification of the tumor microenvironment (TME). This latter process, together with the pro-leukemic polarization of the immune system by interacting with cells both of the innate and of the adaptative immunity (e.g. interacting with T-cell lymphocytes, included Treg, and Natural killer cells), is one of the key mechanisms for immune escape by leukemic cells. The pro-tumorigenic action mentioned above was validated by recent research on EVs produced from CML (86, 87). Additionally, it is known that they can transmit the *BCR::ABL1* transcript, both with and without mutations associated with TKI-resistance (27, 88–90).

**Figure 2:**
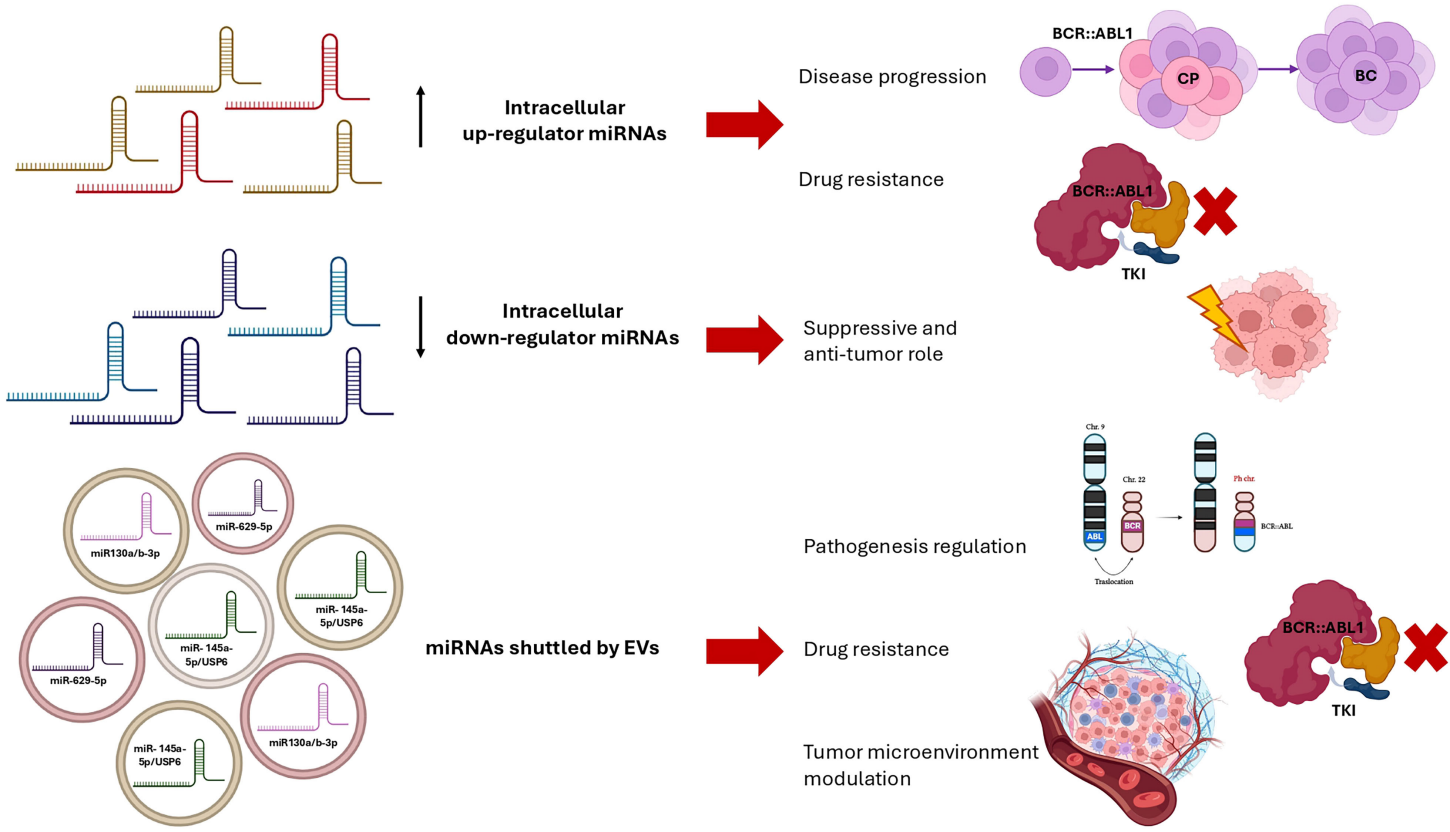
Schematic representation of the main impacts of up-regulated miRNAs, down-regulated miRNAs, and miRNAs shuttled by Extracellular Vesicles in the specific context of Chronic Myeloid Leukemia (miRNA, microRNA; EVs, Extracellular Vesicles; CP, Chronic Phase; BC, Blast Crisis; TKI, Tyrosine Kinase Inhibitor).

In PubMed, 23 publications resulted between 2020 and 2025, 4 of which are out of topic. The search also brought out 1 commentary to the Editor (27)and 5 reviews (88, 91–94). Some of these latter are focused on specific aspects or effects of EVs in CML, underlying the importance of this topic for scientists involved in CML biology and the intense research activity. Moreover, the first application of artificial intelligence (AI) on the effect of exosomes in a mouse xenograft model of CML was published in the considered time frame (95). The exclusive application of Bayesian model to estimate the effect of TKIs, EVs, and TKIs-exosomes mixture in CML is of pivotal importance because this is the evidence of how much info can be derived from EVs analysis and how many effects they can drive in the cells. Among the published research articles, the majority focused on the investigation of how the EVs cargoes influence the response to therapy or may be used to boost the drugs’ efficacy, both when CML cells are the origin or the recipient ones (96). In particular, it has been demonstrated that exosomes derived from a natural killer cell line (NK92MI) are able to support anti-leukemic effect and eliminate different *in vitro* models of hematologic malignancies, including K562 as CML models. Moreover, NK92MI derived exosomes resulted selective against malignant cells by testing their lack of effect on healthy B-cell (97). NK cells have been investigated in parallel in another study, which explored the effect of EVs released by NK3.3 cell lines on solid tumors and CML (K562) cell lines. NK3.3 derived EVs resulted with a cytotoxic effect on K562 imatinib-resistant cells, more than on sensitive K562. In addition, the authors demonstrated that NK3.3 EVs reduced the viability of *in vitro* leukemic stem cells and their expression of leukemia promoting genes (98). On the same *in vitro* model, another group previously demonstrated the impact of vesicular miRNA in the setting of CML by evaluating miR-711 transferred from K562 cell line to BM-MSCs. By performing a co-cultured experiment, vesicular miR-711 decreased adhesive abilities in BM-MSCs by reducing the expression of CD44 (99). In parallel, umbilical cord mesenchymal stem cell-derived EVs have been observed as able to support the apoptosis induced by imatinib in an *in vitro* model, even in resistant cell type, in association with miR-145a-5p/USP6 content. This latter target glutaminase-1 ubiquitination. The results of this study highlight the importance of glutaminase-1 ubiquitination and vesicular miRNA in the setting of therapy resistance in leukemia (100). EVs-mediated regulation of imatinib resistance in CML was related with EVs miRNA cargoes also by another publication just few months ago. This latter research has described the effects of miR-629-5p shuttled by EVs released by imatinib-resistant cells and administered to sensitive ones and observed the activation of the SENP2/PI3K/AKT/mTOR pathway (101). Again, the results confirm that vesicular miRNA are able to drive the TKIs-resistance in CML *in vitro* models and support further investigations, such as the induction of resistance to other therapies. In 2023, an international study corroborated the knowledge about vesicular miRNA impact on therapy-resistance in animal leukemic models by demonstrating how the EVs shuttled miRNA modulates different pathways including those involved in ionizing radiation resistance (**Figure 2**) (102). Finally, EVs shuttled miRNA are the topic of another important study investigating exosomal miR-130a/b-3p and demonstrating its capacity to support the development of a leukemia-favored microenvironment by acting on bone marrow stromal cells, and the subclonal evolution (103). Very similarly, in a leukemic mouse model, leukemic EVs were reported to induce notable transcriptome alterations in Tregs, inhibit mTOR-S6, and trigger STAT5 signaling. This research demonstrates again how leukemic EVs promote the formation of leukemia and immunosuppressive T lymphocytes (104). This last evidence confirms what previously demonstrated by the same group concerning the capability of CML derived EVs to drive Treg lymphocytes via Foxp3 modulation (105). Similarly, K562-derived exosomes were reported to alter human primary cord blood-derived T cells, favoring a leukaemia-promoting microenvironment. Again, *NQO1, PD1*, and *FoxP3* and other genes involved in inhibiting T cells resulted over-expressed after the exposure to CML-derived EVs. In conclusion, these publications suggested that CML cells release EVs that may drive the T cells fates toward malignant-favorable T cells instead of normally activated T cells (106). All together the results published in the last 5 years stressed the importance of EVs analysis in CML to better understand the disease pathogenesis and clinical evolution. Moreover, the data could drive further investigation for the identification of new treatment strategy for myeloid neoplasms targeting EVs cargoes or cell-to-cell communication.

### The dual role of BCR::ABL1 and non BCR::ABL1 mutations: a focus on ASXL1

CML remains a major clinical challenge due to resistance to TKIs, the cornerstone of therapy. The BCR::ABL1 fusion protein, responsible for leukemic transformation, is the target of these TKIs, but mutations in the ATP binding site and myristoyl pocket drive resistance to both first line and novel treatments (107, 108).

ATP binding site mutations, particularly T315I, confer resistance to first- and second-generation TKIs (imatinib, dasatinib, nilotinib), necessitating third-generation inhibitors like ponatinib. Other mutations (E255K/V, F359V/C, M351T, T315A, V299L, Y253H) alter drug binding, reducing efficacy (109–111).

Recently, resistance mutations in the myristoyl pocket have gained attention, particularly affecting asciminib, a novel allosteric inhibitor (112). Asciminib prevents kinase activation by disrupting BCR::ABL1-myriostate interactions, but mutations in this pocket (e.g., F317L/V) impair its efficacy, adding complexity to resistance mechanisms (113–115).

In parallel, additional somatic mutations, especially in *ASXL1* (30–32, 116, 117), *RUNX1* (118–121), *TET2* (33, 122–127), *DNMT3A* (33, 123, 124) and *TP53* (33, 117, 118, 128), significantly influence disease progression (119, 129) and treatment resistance (130–132), even if it is still matter of debate (133).

The prognostic significance of these mutations is complex and sometimes contradictory. For instance, *ASXL1* mutations have been associated with worse event-free survival in chronic phase CML, suggesting a negative impact on prognosis (70). Conversely, some studies indicate that while these mutations are prevalent, their direct influence on treatment outcomes may vary, highlighting the need for further research to clarify their roles (134). Similarly, mutations in *RUNX1* and *TP53* are often linked to disease progression and poor clinical response, yet the extent of their impact can differ between patients (135). This variability underscores the importance of personalized genetic assessment in CML management (136).

These mutations disrupt clonal evolution, creating a complex molecular landscape that affects prognosis and complicates therapy, particularly in advanced stages (30, 116, 118, 137). Many of these mutations occur in genes involved in epigenetic modifications (31, 125, 128), chromatin remodeling (124, 138), and DNA repair (128, 129), which can lead to treatment resistance (118, 139, 140), rapid disease transformation (127, 129, 137), and adverse clinical features such as high leukocyte counts and splenomegaly (34, 129). These acquired mutations, typically in advanced stages, correlate with poor responses to TKIs and higher relapse rates (29, 30, 141). Integrating genetic testing into routine management of CML is crucial for improving risk stratification and identifying patients who might benefit from alternative or more aggressive therapies (32, 35, 118, 142, 143).

Among these mutations, *ASXL1* mutations play a particularly prominent role in CML (30, 118, 121, 125), affecting chromatin remodeling and epigenetic regulation (144), processes essential for maintaining normal cellular differentiation and function. *ASXL1* mutations promote a myeloproliferative phenotype and accelerate the transition to blast phase, complicating disease management (29, 31, 118, 122). Patients with *ASXL1* mutations often present with high-risk features such as elevated leukocyte counts, splenomegaly, and resistance to TKIs, resulting from disruptions in transcriptional repression and DNA methylation pathways (29, 30, 32, 122). Some studies suggest that *ASXL1* mutations increase leukemic cell self-renewal and resistance to differentiation (128), promoting rapid disease progression and reduced TKI efficacy (123). These mutations are increasingly recognized as independent prognostic markers, especially in patients whose disease does not respond well to first-line TKI treatment (32, 118, 119, 139). Identifying *ASXL1* mutations through genetic diagnostics can help in stratifying high-risk patients and informing targeted therapies (122, 127).

A PubMed search resulted in 123 publications from 2020 to 2025, of which 100 were unrelated to CML. Thus, 23 articles were selected for analysis, revealing several key themes regarding the role of mutations in CML, with particular focus on *ASXL1* mutations.

To summarize, the majority of studies analyzed highlight *ASXL1* mutations as significant prognostic biomarkers, consistently linking them to worse event-free survival, increased resistance to TKIs, and disease progression (30–32, 119, 140, 145).

Also in pediatric patients, germline mutations in genes such as *ASXL1* and *NOTCH1* suggest a genetic predisposition that accelerates leukemogenesis (127, 140, 146).

Advanced diagnostic techniques like whole-exome sequencing have revealed a broader mutational landscape, highlighting the potential for expanded diagnostic panels to improve clinical management (117, 128). Complex clinical cases of resistant CML further underscore the role of multiple mutations in driving disease progression and influencing outcomes (119, 139, 141).

## Discussion

This review provided a comprehensive overview of the latest insights into CML biology, emphasizing critical advancements in understanding its pathogenesis, progression, and therapeutic resistance. While CML is generally viewed as a manageable disease with survival rates comparable to the general population (147), significant challenges persist, particularly for patients who develop TKI resistance, relapse, or transition to advanced stages.

Among the key areas of investigation, LSCs stand out for their persistence during treatment-free remission and their potential role in disease relapse and resistance (148). Their quiescent nature and ability to evade both therapeutic and immune responses make them a central challenge in CML management, necessitating deeper exploration into their survival mechanisms and resistance pathways. Understanding how LSCs interact with their niche and maintain dormancy could pave the way for targeted interventions to eliminate these reservoirs of disease.

EVs have emerged as pivotal mediators of intercellular communication, influencing drug resistance, immune evasion, and leukemogenesis. By transferring bioactive cargoes, such as *BCR::ABL1* transcripts and resistance-associated miRNAs, EVs not only facilitate disease progression but also present a promising avenue for therapeutic targeting. However, their heterogeneity and the complexity of their cargoes, which reflect the heterogeneity of patients’ and *in vitro* models’ (149), highlight the need for advanced technologies to better characterize and harness their potential (91).

Furthermore, mutations beyond the primary *BCR::ABL1* fusion gene, particularly in genes such as *ASXL1, RUNX1*, and *TP53*, complicate the molecular landscape of CML. These mutations are strongly linked to poor prognosis, accelerated disease progression, and resistance to TKI therapy. Incorporating comprehensive genetic profiling into routine clinical practice could enhance risk stratification and enable the development of personalized treatment strategies for high-risk patients (150).

Additionally, miRNAs play a dual role in CML, acting as regulators of leukemogenesis, progression, and therapeutic response. Dysregulated miRNAs contribute to resistance mechanisms and promote leukemic stem cell survival. As they are accessible in biological fluids and show potential as therapeutic targets, miRNAs represent a promising area of focus. However, a deeper understanding of their context-dependent roles is essential to translate these findings into clinical applications.

Integrating these insights into CML biology is pivotal for transforming the landscape of disease management. Future efforts must prioritize the development of precision medicine approaches that address the multifaceted challenges posed by LSCs, EVs, mutations, and miRNAs. Nevertheless, we expect that in the near future, analyses related to LSCs and genomic landscape will be more easily translated into clinical practice than those related to EVs and miRNAs. This is both taking into consideration the workflow they require, the speed of interpretation of the resulting information, and the possibility that these same elements may become targets for further targeted therapies.

Advanced technologies, such as single-cell multiomics and targeted therapeutics, could enable the elimination of minimal residual disease and overcome therapy resistance. Achieving these goals requires a multidisciplinary approach that leverages the latest advancements in molecular biology, immunology, and clinical practice. By embracing this integrative strategy, the goal of complete disease eradication and improved patient outcomes becomes increasingly attainable.

## References

[1] Wang Y Liang Zj Gale RP Liao H Ma J Gong Tj. Chronic myeloid leukaemia: Biology and therapy. Blood Rev. (2024) 65:101196. doi: 10.1016/j.blre.2024.101196 38604819

[2] Jabbour E Kantarjian H. Chronic myeloid leukemia: 2025 update on diagnosis, therapy, and monitoring. Am J Hematol. (2024) 99:2191–212. doi: 10.1002/ajh.27443 39093014

[3] Wu A Liu X Fruhstorfer C Jiang X. Clinical insights into structure, regulation, and targeting of ABL kinases in human leukemia. Int J Mol Sci. (2024) 25:3307. doi: 10.3390/ijms25063307 38542279 PMC10970269

[4] Kockerols C Valk PJM Dulucq S Nicolini FE Mahon FX Atallah E. BCR::ABL1 digital PCR for treatment-free remission prediction in chronic myeloid leukemia patients: An individual participant data meta-analysis. Am J Hematol. (2024) 99:1632–5. doi: 10.1002/ajh.27359 38769689

[5] Zanaglio C Bernardi S Gandolfi L Farina M Re F Polverelli N. RT-qPCR versus digital PCR: how do they impact differently on clinical management of chronic myeloid leukemia patients? Case Rep Oncol. (2020) 13:1263–9. doi: 10.1159/000510440 PMC7670369 33250741

[6] Malagola M Iurlo A Abruzzese E Bonifacio M Stagno F Binotto G. Molecular response and quality of life in chronic myeloid leukemia patients treated with intermittent TKIs: First interim analysis of OPTkIMA study. Cancer Med. (2021) 10:1726–37. doi: 10.1002/cam4.v10.5 PMC7940223 33594821

[7] Soverini S Bernardi S Galimberti S. Molecular testing in CML between old and new methods: are we at a turning point? J Clin Med. (2020) 9:3865. doi: 10.3390/jcm9123865 33261150 PMC7760306

[8] Malagola M Iurlo A Bucelli C Abruzzese E Bonifacio M Stagno F. The italian multicentric randomized OPTkIMA trial on fixed vs progressive intermittent TKI therapy in CML elderly patients: 3-years of molecular response and quality of life monitoring after completing the treatment plan. Clin Lymphoma Myeloma Leuk. (2024) 24:323–31. doi: 10.1016/j.clml.2024.01.008 38369436

[9] Kockerols CCB Valk PJM Levin MD Pallisgaard N Cornelissen JJ Westerweel PE. Digital PCR for BCR-ABL1 quantification in CML: current applications in clinical practice. Hemasphere. (2020) 4:e496. doi: 10.1097/HS9.0000000000000496 33283168 PMC7710259

[10] Bernardi S Cavalleri A Mutti S Garuffo L Farina M Leoni A. Digital PCR (dPCR) is able to anticipate the achievement of stable deep molecular response in adult chronic myeloid leukemia patients: results of the DEMONSTRATE study. Ann Hematol. (2024) 104(1):207–17. doi: 10.1007/s00277-024-06100-4 PMC11868186 39611878

[11] Russo D Garcia-gutierrez JV Soverini S Baccarani M. Chronic myeloid leukemia prognosis and therapy: criticisms and perspectives. J Clin Med. (2020) 9:1709. doi: 10.3390/jcm9061709 32498406 PMC7357035

[12] Hochhaus A Baccarani M Silver RT Schiffer C Apperley JF Cervantes F. European LeukemiaNet 2020 recommendations for treating chronic myeloid leukemia. Leukemia. (2020) 34:966–84. doi: 10.1038/s41375-020-0776-2 PMC7214240 32127639

[13] Russo D Malagola M Polverelli N Farina M Re F Bernardi S. Twenty years of evolution of CML therapy: how the treatment goal is moving from disease to patient. Ther Adv Hematol. (2023) 14:20406207231216077. doi: 10.1177/20406207231216077 38145059 PMC10748527

[14] Yeung DT Shanmuganathan N Reynolds J Branford S Walia M Yong ASM. Asciminib monotherapy as frontline treatment of chronic-phase chronic myeloid leukemia: results from the ASCEND study. Blood. (2024) 144(19):1993–2001. doi: 10.1182/blood.2024024657 39102630

[15] Ram M Afrash MR Moulaei K Parvin M Esmaeeli E Karbasi Z. Application of artificial intelligence in chronic myeloid leukemia (CML) disease prediction and management: a scoping review. BMC Cancer. (2024) 24:1026. doi: 10.1186/s12885-024-12764-y 39164653 PMC11337640

[16] Sasaki K Jabbour EJ Ravandi F Konopleva M Borthakur G Wierda WG. The LEukemia Artificial Intelligence Program (LEAP) in chronic myeloid leukemia in chronic phase: A model to improve patient outcomes. Am J Hematol. (2020) 96:241. doi: 10.1002/ajh.26047 33180322 PMC9022629

[17] Bernardi S Vallati M Gatta R. Artificial intelligence-based management of adult chronic myeloid leukemia: where are we and where are we going? Cancers (Basel). (2024) 16:848. doi: 10.3390/cancers16050848 38473210 PMC10930728

[18] de Oliveira FM Jamur VR Merfort LW Pozzo AR Mai S. Three-dimensional nuclear telomere architecture and differential expression of aurora kinase genes in chronic myeloid leukemia to measure cell transformation. BMC Cancer. (2022) 22(1):1024. doi: 10.1186/s12885-022-10094-5 36175852 PMC9520804

[19] Telliam G Desterke C Imeri J M’kacher R Oudrhiri N Balducci E. Modeling global genomic instability in chronic myeloid leukemia (CML) using patient-derived induced pluripotent stem cells (iPSCs). Cancers (Basel). (2023) 15:2594. doi: 10.3390/cancers15092594 37174060 PMC10177163

[20] Rinaldi I Winston K. Chronic myeloid leukemia, from pathophysiology to treatment-free remission: A narrative literature review. J Blood Med. (2023) 14:261. doi: 10.2147/JBM.S382090 37051025 PMC10084831

[21] Yohanan B George B. Current management of chronic myeloid leukemia myeloid blast phase. Clin Med Insights Oncol. (2022) 16:11795549221139356. doi: 10.1177/11795549221139357 PMC9726842 36507316

[22] Sobhia ME Kumar GS Mallick A Singh H Kumar K Chaurasiya M. Computational and biological investigations on abl1 tyrosine kinase: A review. Curr Drug Targets. (2021) 22:38–51. doi: 10.2174/1389450121999201013152513 33050861

[23] Abruzzese E Bocchia M Trawinska MM Raspadori D Bondanini F Sicuranza A. Minimal Residual Disease Detection at RNA and Leukemic Stem Cell (LSC) Levels: Comparison of RT-qPCR, d-PCR and CD26+ Stem Cell Measurements in Chronic Myeloid Leukemia (CML) Patients in Deep Molecular Response (DMR). Cancers (Basel). (2023) 15:4112. doi: 10.3390/cancers15164112 37627140 PMC10452239

[24] Klümper T Bruckmueller H Diewock T Kaehler M Haenisch S Pott C. Expression differences of miR-142-5p between treatment-naïve chronic myeloid leukemia patients responding and non-responding to imatinib therapy suggest a link to oncogenic ABL2, SRI, cKIT and MCL1 signaling pathways critical for development of therapy resistance. Exp Hematol Oncol. (2020) 9:26. doi: 10.1186/s40164-020-00183-1 32999756 PMC7519530

[25] Jurkovicova D Lukackova R Magyerkova M Kulcsar L Krivjanska M Krivjansky V. microRNA expression profiling as supportive diagnostic and therapy prediction tool in chronic myeloid leukemia. Neoplasma. (2015) 62(6):949–58. doi: 10.4149/neo_2015_115 26458312

[26] Srutova K Curik N Burda P Savvulidi F Silvestri G Trotta R. BCR-ABL1 mediated miR-150 downregulation through MYC contributed to myeloid differentiation block and drug resistance in chronic myeloid leukemia. Haematologica. (2018) 103(12):2016–25. doi: 10.3324/haematol.2018.193086 PMC6269310 30049824

[27] Bernardi S Malagola M Polverelli N Russo D. Exosomes in chronic myeloid leukemia: are we reading a new reliable message? Acta Haematol. (2020) 143(5):509–10. doi: 10.1159/000505088 31922494

[28] Bernardi S Farina M Bosio K Di Lucanardo A Leoni A Re F. Feasibility of leukemia-derived exosome enrichment and co-isolated dsDNA sequencing in acute myeloid leukemia patients: A proof of concept for new leukemia biomarkers detection. Cancers (Basel). (2022) 14:4504. doi: 10.3390/cancers14184504 36139664 PMC9497185

[29] Breccia M. Atypical CML: diagnosis and treatment. Hematol Am Soc Hematol Educ Program. (2023) 2023:476–82. doi: 10.1182/hematology.2023000448 PMC10727105 38066919

[30] Bidikian A Kantarjian H Jabbour E Short NJ Patel K Ravandi F. Prognostic impact of ASXL1 mutations in chronic phase chronic myeloid leukemia. Blood Cancer J. (2022) 12:144. doi: 10.1038/s41408-022-00742-1 36307398 PMC9616867

[31] Schönfeld L Rinke J Hinze A Nagel SN Schäfer V Schenk T. ASXL1 mutations predict inferior molecular response to nilotinib treatment in chronic myeloid leukemia. Leukemia. (2022) 36:2242. doi: 10.1038/s41375-022-01648-4 35902731 PMC9417980

[32] Rafiq Mohammed A Assad D Rostami G Hamid M. Frequency and prognostic influence of ASXL1 mutations and its potential association with BCR-ABL1 transcript type and smoke in chronic myeloid leukemia patients. Gene. (2023) 886:147776. doi: 10.1016/j.gene.2023.147776 37689224

[33] Guerineau H Cayuela J Dulucq S Tran Quang V Tarfi S Gricourt G. Mutation of epigenetic regulators at diagnosis is an independent predictor of tyrosine kinase inhibitor treatment failure in chronic myeloid leukemia: A report from the RESIDIAG study. Am J Hematol. (2025) 100:507–10. doi: 10.1002/ajh.v100.3 39654506

[34] Romzova M Smitalova D Hynst J Tom N Loja T Herudkova Z. Hierarchical distribution of somatic variants in newly diagnosed chronic myeloid leukaemia at diagnosis and early follow-up. Br J Haematol. (2021) 194:604–12. doi: 10.1111/bjh.v194.3 34212373

[35] Imataki O Ishida T Kubo H Uemura M Nanya Y Kawakami K. A case of tyrosine kinase inhibitor-resistant chronic myeloid leukemia, chronic phase with ASXL1 mutation. Case Rep Oncol. (2020) 13:449–55. doi: 10.1159/000506452 PMC7204851 32399015

[36] Buss EC Ho AD. Leukemia stem cells. Int J Cancer. (2011) 129:2328–36. doi: 10.1002/ijc.26318 21796620

[37] Corbin AS Agarwal A Loriaux M Cortes J Deininger MW Druker BJ. Human chronic myeloid leukemia stem cells are insensitive to imatinib despite inhibition of BCR-ABL activity. J Clin Invest. (2010) 121:396. doi: 10.1172/JCI35721 21157039 PMC3007128

[38] Galimberti S Grassi S Baratè C Guerrini F Ciabatti E Perutelli F. The polycomb BMI1 protein is co-expressed with CD26+ in leukemic stem cells of chronic myeloid leukemia. Front Oncol. (2018) 8:555. doi: 10.3389/fonc.2018.00555 30574454 PMC6291509

[39] Sicuranza A Raspadori D Bocchia M. CD26/DPP-4 in chronic myeloid leukemia. Cancers (Basel). (2022) 14:891. doi: 10.3390/cancers14040891 35205639 PMC8870104

[40] Valent P Sadovnik I Ráčil Z Herrmann H Blatt K Cerny-Reiterer S. DPPIV (CD26) as a novel stem cell marker in Ph+ chronic myeloid leukaemia. Eur J Clin Invest. (2014) 44:1239–45. doi: 10.1111/eci.12368 25371066

[41] Herrmann H Sadovnik I Cerny-Reiterer S Rülicke T Stefanzl G Willmann M. (CD26) defines leukemic stem cells (LSC) in chronic myeloid leukemia. Blood. (2014) 123:3951–62. doi: 10.1182/blood-2013-10-536078 24778155

[42] Raspadori D Pacelli P Sicuranza A Abruzzese E Iurlo A Cattaneo D. Flow cytometry assessment of CD26+ Leukemic stem cells in peripheral blood: A simple and rapid new diagnostic tool for chronic myeloid leukemia. Cytomet B Clin Cytom. (2019) 96:294. doi: 10.1002/cyto.b.21764 PMC6767040 30714299

[43] Bocchia M Sicuranza A Abruzzese E Iurlo A Sirianni S Gozzini A. Residual peripheral blood CD26+ Leukemic stem cells in chronic myeloid leukemia patients during TKI therapy and during treatment-free remission. Front Oncol. (2018) 8:194. doi: 10.3389/fonc.2018.00194 29900128 PMC5988870

[44] Sicuranza A Santoni A Pacelli P Freducci S Pacini E Abruzzese E. FATE and role of peripheral blood CD26+ Leukemia STEM CELLS at diagnosis in chronic myeloid leukemia patients: FINAL results of prospective flowers study. Blood. (2024) 144:995–5. doi: 10.1182/blood-2024-204689

[45] Pacelli P Santoni A Sicuranza A Abruzzese E Giai V Crugnola M. Prospective monitoring of chronic myeloid leukemia patients from the time of TKI discontinuation: the fate of peripheral blood CD26+ leukemia stem cells. Front Pharmacol. (2023) 14:1194712. doi: 10.3389/fphar.2023.1194712 37305536 PMC10250640

[46] Warfvinge R Ulfsson LG Dhapola P Safi F Sommarin M Soneji S. Single-cell multiomics analysis of chronic myeloid leukemia links cellular heterogeneity to therapy response. Elife. (2024) 12:RP92074. doi: 10.7554/eLife.92074.3.sa3 39503729 PMC11540304

[47] Komic H Nilsson MS Wennström L Bandaru TS Jaako P Hellstrand K. Single-cell proteo-transcriptomic profiling reveals altered characteristics of stem and progenitor cells in patients receiving cytoreductive hydroxyurea in early-phase chronic myeloid leukemia. Haematologica. (2024) 110:117. doi: 10.3324/haematol.2024.285071 PMC11694111 39157872

[48] Lahlil R Aries A Scrofani M Zanetti C Hennequin D Drénou B. Stem cell responsiveness to imatinib in chronic myeloid leukemia. Int J Mol Sci. (2023) 24:16671. doi: 10.3390/ijms242316671 38068992 PMC10706348

[49] Wang J Ma W Huang J Qiu G Zhang T Wei Q. HIF-2α inhibition disrupts leukemia stem cell metabolism and impairs vascular microenvironment to enhance chronic myeloid leukemia treatment. Cancer Lett. (2024) 597:217060. doi: 10.1016/j.canlet.2024.217060 38880225

[50] Chai J Wang Q Qiu Q Han G Chen Y Li W. YBX1 regulates the survival of chronic myeloid leukemia stem cells by modulating m6A-mediated YWHAZ stability. Cell Oncol. (2023) 46:451–64. doi: 10.1007/s13402-022-00762-w PMC12974669 36512307

[51] Scott MT Liu W Mitchell R Clarke CJ Kinstrie R Warren F. Activating p53 abolishes self-renewal of quiescent leukaemic stem cells in residual CML disease. Nat Commun. (2024) 15:651. doi: 10.1038/s41467-024-44771-9 38246924 PMC10800356

[52] Filik Y Bauer K Hadzijusufovic E Haider P Greiner G Witzeneder N. PI3-kinase inhibition as a strategy to suppress the leukemic stem cell niche in Ph+ chronic myeloid leukemia. Am J Cancer Res. (2021) 11:6042. 35018241 PMC8727792

[53] Rui SHI Xinle LIU. CD44: a potential therapeutic target in chronic myeloid leukemia. Pharmazie. (2021) 76:574–8. doi: 10.1691/ph.2021.1744 34986951

[54] Ruiz MS Sánchez MB Bonecker S Furtado C Koile D Yankilevich P. miRNome profiling of LSC-enriched CD34+CD38–CD26+ fraction in Ph+ CML-CP samples from Argentinean patients: a potential new pharmacogenomic tool. Front Pharmacol. (2021) 11:612573. doi: 10.3389/fphar.2020.612573 33569005 PMC7869017

[55] Bartel DP. MicroRNAs: genomics, biogenesis, mechanism, and function. Cell. (2004) 116:281–97. doi: 10.1016/S0092-8674(04)00045-5 14744438

[56] Cipolla GA. A non-canonical landscape of the microRNA system. Front Genet. (2014) 5:337. doi: 10.3389/fgene.2014.00337 25295056 PMC4172024

[57] Liu X Fortin K Mourelatos Z. MicroRNAs: biogenesis and molecular functions. Brain Pathol. (2008) 18:113. doi: 10.1111/j.1750-3639.2007.00121.x 18226106 PMC8095604

[58] Kosaka N Iguchi H Ochiya T. Circulating microRNA in body fluid: a new potential biomarker for cancer diagnosis and prognosis. Cancer Sci. (2010) 101:2087. doi: 10.1111/j.1349-7006.2010.01650.x 20624164 PMC11159200

[59] Kato M Slack FJ. microRNAs: small molecules with big roles –C. elegans to human cancer. Biol Cell. (2008) 100:71–81. doi: 10.1042/BC20070078 18199046

[60] Otmani K Lewalle P. Tumor suppressor miRNA in cancer cells and the tumor microenvironment: mechanism of deregulation and clinical implications. Front Oncol. (2021) 11:708765. doi: 10.3389/fonc.2021.708765 34722255 PMC8554338

[61] Elias MH Syed Mohamad SF Abdul Hamid N. A systematic review of candidate miRNAs, its targeted genes and pathways in chronic myeloid leukemia–an integrated bioinformatical analysis. Front Oncol. (2022) 12:848199. doi: 10.3389/fonc.2022.848199 35330714 PMC8940286

[62] Chavaro-Francisco G Hernández-Zavala A Bravo-Cidro CE Rios-Rodriguez S Muciño-Sánchez M López-López M. Gene variants in components of the microRNA processing pathway in chronic myeloid leukemia. Genes (Basel). (2024) 15:1054. doi: 10.3390/genes15081054 39202414 PMC11353722

[63] Wosniaki DK Marin AM Oliveira RN Koerich GM Munhoz EC Farias JS de H. The screening of microRNAs in chronic myeloid leukemia: A clinical evaluation. Int J Mol Sci. (2024) 25:3363. doi: 10.3390/ijms25063363 38542337 PMC10969883

[64] Parsa-Kondelaji M Musavi M Barzegar F Abbasian N Rostami M Seyedtaghia MR. Dysregulation of miRNA expression in patients with chronic myelogenous leukemia at diagnosis: A systematic review. Biomark Med. 2023. 17:1021–9. doi: 10.2217/bmm-2023-0575 38230979

[65] Bansal M Ansari S Verma M. Role of miRNAs to control the progression of Chronic Myeloid Leukemia by their expression levels. Med Oncol. (2024) 41:55. doi: 10.1007/s12032-023-02278-1 38216843

[66] Navabi A Akbari B Abdalsamadi M Naseri SS. The role of microRNAs in the development, progression and drug resistance of chronic myeloid leukemia and their potential clinical significance. Life Sci. (2022) 296:120437. doi: 10.1016/j.lfs.2022.120437 35231484

[67] Singh R Ha SE Yu TY Ro S. Dual Roles of miR-10a-5p and miR-10b-5p as Tumor Suppressors and Oncogenes in Diverse Cancers. Int J Mol Sci. (2025) 26:415. doi: 10.3390/ijms26010415 39796267 PMC11720153

[68] Chen D Wu D Shao K Ye B Huang J Gao Y. MiR-15a-5p negatively regulates cell survival and metastasis by targeting CXCL10 in chronic myeloid leukemia. Am J Transl Res. (2017) 9:4308–16. PMC5622273 28979704

[69] Akidan O Petrovic N Misir S. mir-188-5p emerges as an oncomir to promote chronic myeloid leukemia via upregulation of BUB3 and SUMO2. Mol Biol Rep. (2025) 52:269. doi: 10.1007/s11033-025-10359-9 40019654

[70] Abdulmawjood B Costa B Roma-rodrigues C Baptista PV Fernandes AR. Genetic biomarkers in chronic myeloid leukemia: what have we learned so far? Int J Mol Sci. (2021) 22:12516. doi: 10.1016/j.omtn.2023.04.026 34830398 PMC8626020

[71] Su R Li C Wang X Li Z Wen Z Yin Z. PPFIA1- targeting miR-181a mimic and saRNA overcome imatinib resistance in BCR-ABL1-independent chronic myeloid leukemia by suppressing leukemia stem cell regeneration. Mol Ther Nucleic Acids. (2023) 32:729–42. doi: 10.1016/j.omtn.2023.04.026 PMC10208829 37234746

[72] Liu X Cui MM Zhu HZ Fu PY Wang GC Huang L. MiR-199a-3p overexpression suppressed cell proliferation and sensitized chronic myeloid leukaemia cells to imatinib by inhibiting mTOR signalling. Acta Haematol. (2022) 145:484–98. doi: 10.1159/000524158 35313299

[73] Wu YY Lai HF Huang TC Chen YG Ye RH Chang PY. Aberrantly reduced expression of miR-342-5p contributes to CCND1-associated chronic myeloid leukemia progression and imatinib resistance. Cell Death Dis. (2021) 12:908. doi: 10.1038/s41419-021-04209-2 34611140 PMC8492784

[74] Mirza MAB Guru SA Abdullah SM Rizvi A Saxena A. microRNA-21 expression as prognostic and therapeutic response marker in chronic myeloid leukaemia patients. Asian Pac J Cancer Prev. (2019) 20:2379–83. doi: 10.31557/APJCP.2019.20.8.2379 PMC6852824 31450909

[75] Habib EM Nosiar NA Eid MA Taha AM Sherief DE Hassan AE. Circulating miR-146a expression predicts early treatment response to imatinib in adult chronic myeloid leukemia. J Invest Med. (2021) 69:333–7. doi: 10.1136/jim-2020-001563 33172871

[76] Ali Beg MM Guru SA Abdullah SM Ahmad I Rizvi A Akhter J. Regulation of miR-126 and miR-122 Expression and Response of Imatinib Treatment on Its Expression in Chronic Myeloid Leukemia Patients. Oncol Res Treat. (2021) 44:530–7. doi: 10.1159/000518722 34515193

[77] Guo Yg Zhang L Hu P Li Zz Zhang R Lv X. Correlation analysis of bone marrow microvessel density and miRNA expression on drug resistance in patients with chronic myelogenous leukemia after tyrosine kinase inhibitor treatment. Hematology. (2024) 29(1):2304488. doi: 10.1080/16078454.2024.2304488 38299685

[78] Qi S Huang J Long R. The synergistic effect of miR-203 and cytarabine on the inhibition of cell proliferation and induction of apoptosis in chronic myelogenous leukemia cells. Pak J Pharm Sci. (2024) 37(5):1019–25. 39460968

[79] McIntyre G Jackson Z Colina J Sekhar S DiFeo A. miR-181a: regulatory roles, cancer-associated signaling pathway disruptions, and therapeutic potential. Expert Opin Ther Targets. (2024) 28(12):1061–91. doi: 10.1080/14728222.2024.2433687 PMC12054384 39648331

[80] Anelli L Zagaria A Specchia G Musto P Albano F. Dysregulation of miRNA in Leukemia: Exploiting miRNA Expression Profiles as Biomarkers. Int J Mol Sci. (2021) 22(13):7156. doi: 10.3390/ijms22137156 34281210 PMC8269043

[81] Wu SC Lai SW Lu XJ Lai HF Chen YG Chen PH. Profiling of miRNAs and their interfering targets in peripheral blood mononuclear cells from patients with chronic myeloid leukaemia. Front Oncol. (2023) 13. doi: 10.3389/fonc.2023.1173970 PMC10356106 37476380

[82] Bernardi S Balbi C. Extracellular vesicles: from biomarkers to therapeutic tools. Biol (Basel). (2020) 9:1–6. doi: 10.3390/biology9090258 PMC7564466 32878063

[83] Bernardi S Zanaglio C Farina M Polverelli N Malagola M Russo D. dsDNA from extracellular vesicles (EVs) in adult AML. Ann Hematol. (2020) 100:1355. doi: 10.1007/s00277-020-04109-z 32474620 PMC8043941

[84] Bernardi S Mulas O Mutti S Costa A Russo D La Nasa G. Extracellular vesicles in the Chronic Myeloid Leukemia scenario: an update about the shuttling of disease markers and therapeutic molecules. Front Oncol. (2023) 13:1239042. doi: 10.3389/fonc.2023.1239042 38260856 PMC10800789

[85] Bernardi S Farina M. Exosomes and extracellular vesicles in myeloid neoplasia: the multiple and complex roles played by these “Magic bullets. Biol (Basel). (2021) 10:105. doi: 10.3390/biology10020105 PMC7912829 33540594

[86] Hrdinova T Toman O Dresler J Klimentova J Salovska B Pajer P. Exosomes released by imatinib-resistant K562 cells contain specific membrane markers, IFITM3, CD146 and CD36 and increase the survival of imatinib-sensitive cells in the presence of imatinib. Int J Oncol. (2021) 58:238–50. doi: 10.3892/ijo.2020.5163 33491750

[87] Fu FF Zhu XJ Wang HX Zhang LM Yuan GL Chen ZC. BCR-ABL1-positive microvesicles Malignantly transform human bone marrow mesenchymal stem cells *in vitro* . Acta Pharmacol Sin. (2017) 38:1475–85. doi: 10.1038/aps.2017.116 PMC5672071 28836580

[88] Jurj A Pasca S Teodorescu P Tomuleasa C Berindan-Neagoe I. Basic knowledge on bcr-abl1-positive extracellular vesicles. Biomark Med. (2020) 14:451–8. doi: 10.2217/bmm-2019-0510 32270699

[89] Bernardi S Foroni C Zanaglio C Re F Polverelli N Turra A. Feasibility of tumor-derived exosome enrichment in the onco-hematology leukemic model of chronic myeloid leukemia. Int J Mol Med. (2019) 44:2133. doi: 10.3892/ijmm.2019.4372 31638195 PMC6844640

[90] Bernardi S Mutti S Cavalleri A Ielo C Farina M Re F. Correlation between BCR::ABL1 transcript LEVEL in circulating extracellular vesicles and BOTH the molecular response and the ongoing therapy: A study on adult CML patients. Blood. (2023) 142:3180–0. doi: 10.1182/blood-2023-185733

[91] Bernardi S Mulas O Mutti S Costa A Russo D La Nasa G. Extracellular vesicles in the Chronic Myeloid Leukemia scenario: an update about the shuttling of disease markers and therapeutic molecules. Front Oncol. (2024) 13:1239042. doi: 10.3389/fonc.2023.1239042 38260856 PMC10800789

[92] Yu M Jin Y Yuan K Liu B Zhu N Zhang K. Effects of exosomes and inflammatory response on tumor: a bibliometrics study and visualization analysis via CiteSpace and VOSviewer. J Cancer Res Clin Oncol. (2024) 150:405. doi: 10.1007/s00432-024-05915-y 39210153 PMC11362500

[93] Swatler J Turos-korgul L Kozlowska E Piwocka K. Immunosuppressive cell subsets and factors in myeloid leukemias. Cancers (Basel). (2021) 13:1203. doi: 10.3390/cancers13061203 33801964 PMC7998753

[94] Cetin Z Saygili EI Yilmaz M. Crosstalk between CML cells with HUVECs and BMSCs through exosomes. Front Biosci - Landmark. (2021) 26:444–67. doi: 10.2741/4901 33049677

[95] Martínez-Fonseca R Vargas-De-León C Reyes-Carreto R Godínez-Jaimes F Martínez-Fonseca R Vargas-De-León C. Bayesian analysis of the effect of exosomes in a mouse xenograft model of chronic myeloid leukemia. Math Biosci Eng. (2023) 20:19504–26. doi: 10.3934/mbe.2023864 38052612

[96] Nikravesh F Mirzaee Khalilabadi R Farsinejad A Mardani Valandani H. Platelet microparticles influence gene expression and modulate biological activities of chronic myeloid leukemia cells (K562). Mol Biol Rep. (2024) 51:1–12. doi: 10.1007/s11033-024-09383-y 38796661

[97] Samara A Anbar M Shapira S Zemlyansky A Zozovsky A Raanani P. Using natural killer cell-derived exosomes as a cell-free therapy for leukemia. Hematol Oncol. (2023) 41:487–98. doi: 10.1002/hon.3111 36451254

[98] McCune A Kornbluth J. NK3.3-derived extracellular vesicles penetrate and selectively kill treatment-resistant tumor cells. Cancers (Basel). (2024) 16:90. doi: 10.3390/cancers16010090 PMC10778188 38201518

[99] Jiang YH Liu J Lin J Li SQ Xu YM Min QH. K562 cell-derived exosomes suppress the adhesive function of bone marrow mesenchymal stem cells via delivery of miR-711. Biochem Biophys Res Commun. (2020) 521:584–9. doi: 10.1016/j.bbrc.2019.10.096 31677790

[100] Chen X Chen Y Zhang M Cheng H Mai H Yi M. HucMSC exosomes promoted imatinib-induced apoptosis in K562-R cells via a miR-145a-5p/USP6/GLS1 axis. Cell Death Dis. (2022) 13:92. doi: 10.1038/s41419-022-04531-3 35091542 PMC8799639

[101] Jiang Y Xiao S Huang S Zhao X Ding S Huang Q. Extracellular vesicle-mediated regulation of imatinib resistance in chronic myeloid leukemia via the miR-629-5p/SENP2/PI3K/AKT/mTOR axis. Hematology. (2024) 29(1):92. doi: 10.1080/16078454.2024.2379597 39056503

[102] Csordás IB Rutten EA Szatmári T Subedi P Cruz-Garcia L Kis D. The miRNA content of bone marrow-derived extracellular vesicles contributes to protein pathway alterations involved in ionising radiation-induced bystander responses. Int J Mol Sci. (2023) 24:8607. doi: 10.3390/ijms24108607 37239971 PMC10218377

[103] Chai C Sui K Tang J Yu H Yang C Zhang H. BCR-ABL1-driven exosome-miR130b-3p-mediated gap-junction Cx43 MSC intercellular communications imply therapies of leukemic subclonal evolution. Theranostics. (2023) 13:3943. doi: 10.7150/thno.83178 37554265 PMC10405834

[104] Swatler J Turos-Korgul L Brewinska-Olchowik M De Biasi S Dudka W Le BV. 4-1BBL–containing leukemic extracellular vesicles promote immunosuppressive effector regulatory T cells. Blood Adv. (2022) 6:1879. doi: 10.1182/bloodadvances.2021006195 35130345 PMC8941461

[105] Swatler J Dudka W Bugajski L Brewinska-Olchowik M Kozlowska E Piwocka K. Chronic myeloid leukemia-derived extracellular vesicles increase Foxp3 level and suppressive activity of thymic regulatory T cells. Eur J Immunol. (2019) 50:606. doi: 10.1002/eji.201848051 31758697 PMC7187374

[106] Jafarzadeh N Gholampour MA Alivand MR Kavousi S Arzi L Rad F. CML derived exosomes promote tumor favorable functional performance in T cells. BMC Cancer. (2021) 21:1002. doi: 10.1186/s12885-021-08734-3 34493241 PMC8424959

[107] Teng M Luskin MR Cowan-Jacob SW Ding Q Fabbro D Gray NS. The dawn of allosteric BCR-ABL1 drugs: from a phenotypic screening hit to an approved drug. J Med Chem. (2022) 65:7581–94. doi: 10.1021/acs.jmedchem.2c00373 35609336

[108] George B Chan KH Rios A. Therapeutic options for chronic myeloid leukemia following the failure of second-generation tyrosine kinase inhibitor therapy. Front Oncol. (2024) 14:1446517. doi: 10.3389/fonc.2024.1446517 39139284 PMC11320603

[109] Tachibana T Kondo T Uchida N Doki N Takada S Takahashi S. The clinical significance of BCR-ABL1 mutations in patients with philadelphia chromosome–positive chronic myeloid leukemia who underwent allogeneic hematopoietic cell transplantation. Transplant Cell Ther. (2022) 28:321.e1–8. doi: 10.1016/j.jtct.2022.03.009 35296447

[110] Sánchez R Dorado S Ruíz-Heredia Y Martín-Muñoz A Rosa-Rosa JM Ribera J. Detection of kinase domain mutations in BCR::ABL1 leukemia by ultra-deep sequencing of genomic DNA. Sci Rep. (2022) 12:13057. doi: 10.1038/s41598-022-17271-3 35906470 PMC9338264

[111] Liu J Yang H Xu X Yi S Meng L. Mutations in the BCR-ABL1 kinase domain in patients with chronic myeloid leukaemia treated with TKIs or at diagnosis. Oncol Lett. (2020) 20:1071. doi: 10.3892/ol.2020.11650 32724345 PMC7377099

[112] Deeks ED. Asciminib: first approval. Drugs. (2022) 82:219–26. doi: 10.1007/s40265-021-01662-3 35041175

[113] Padala S Cortes J. Asciminib in chronic myeloid leukemia: a STAMP for expedited delivery? Haematologica. (2023) 108:2913. doi: 10.3324/haematol.2022.282361 37102603 PMC10620583

[114] Réa D Hughes TP. Development of asciminib, a novel allosteric inhibitor of BCR-ABL1. Crit Rev Oncol Hematol. (2022) 171:103580. doi: 10.1016/j.critrevonc.2022.103580 35021069

[115] Costa A Scalzulli E Bisegna ML Breccia M. Asciminib in the treatment of chronic myeloid leukemia in chronic phase. Future Oncol. (2025) 21(7):815–31. doi: 10.1080/14796694.2025.2464494 PMC11921165 39936231

[116] Ernst T Busch M Rinke J Ernst J Haferlach C Beck JF. Frequent ASXL1 mutations in children and young adults with chronic myeloid leukemia. Leukemia. (2018) 32:2046–9. doi: 10.1038/s41375-018-0157-2 29899367

[117] Menezes J Salgado RN Acquadro F Gómez-Ló Pez G Carralero MC Barroso A. ASXL1, TP53 and IKZF3 mutations are present in the chronic phase and blast crisis of chronic myeloid leukemia. Blood Cancer J. (2013) 3:e157. doi: 10.1038/bcj.2013.54 24212482 PMC3880437

[118] Ochi Y. Genetic landscape of chronic myeloid leukemia. Int J Hematol. (2023) 117:30–6. doi: 10.1007/s12185-022-03510-w 36477676

[119] Oyogoa E Streich L Raess PW Braun T. Case Report: ASXL1, RUNX1, and IDH1 mutation in tyrosine kinase-independent resistant chronic myeloid leukemia progressing to chronic myelomonocytic leukemia-like accelerated phase. Front Oncol. (2023) 13:1217153. doi: 10.3389/fonc.2023.1217153 37746298 PMC10513384

[120] Ochi Y. Clinical significance of clonal evolution in chronic myeloid leukemia. Rinsho Ketsueki. (2023) 64:369–75. doi: 10.11406/rinketsu.64.369 37271527

[121] Miyashita N Onozawa M Kasahara K Matsukawa T Onodera Y Suzuki K. CML with mutant ASXL1 showed decreased sensitivity to TKI treatment via upregulation of the ALOX5-BLTR signaling pathway. Cancer Sci. (2025) 0:1–11. doi: 10.1111/cas.70007 PMC11967257 39905783

[122] Wu W Xu N Zhou X Liu L Tan Y Luo J. Integrative genomic analysis reveals cancer-associated gene mutations in chronic myeloid leukemia patients with resistance or intolerance to tyrosine kinase inhibitor. Onco Targets Ther. (2020) 13:8581–91. doi: 10.2147/OTT.S257661 PMC7468532 32943879

[123] Rinke J Hochhaus A Ernst T. CML - Not only BCR-ABL1 matters. Best Pract Res Clin Haematol. (2020) 33(3):101194. doi: 10.1016/j.beha.2020.101194 33038988

[124] Sant’Antonio E Camerini C Rizzo V Musolino C Allegra A. Genetic heterogeneity in chronic myeloid leukemia: how clonal hematopoiesis and clonal evolution may influence prognosis, treatment outcome, and risk of cardiovascular events. Clin Lymphoma Myeloma Leuk. (2021) 21:573–9. doi: 10.1016/j.clml.2021.04.014 34078586

[125] Chaudhary P Chaudhary S Patel F Patel S Patel D Patel L. Significance of somatic mutation profiling in CML beyond BCR-ABL: A retrospective study of the Indian population. Indian J Hematol Blood Transfus. (2025) 41(1):10–22. doi: 10.1007/s12288-024-01808-9 PMC11794774 39917513

[126] Moncada A Pancrazzi A. Lab tests for MPN. Int Rev Cell Mol Biol. (2022) 366:187–220. doi: 10.1016/bs.ircmb.2021.02.010 35153004

[127] Krumbholz M Dolnik A Sträng E Ghete T Skambraks S Hutter S. A high proportion of germline variants in pediatric chronic myeloid leukemia. Mol Cancer. (2024) 23:206. doi: 10.1186/s12943-024-02109-5 39327604 PMC11426096

[128] Kazemi-Sefat GE Keramatipour M Vaezi M Razavi SM Kavousi K Talebi A. Integrated genomic sequencing in myeloid blast crisis chronic myeloid leukemia (MBC-CML), identified potentially important findings in the context of leukemogenesis model. Sci Rep. (2022) 12:12816. doi: 10.1038/s41598-022-17232-w 35896598 PMC9329277

[129] Alanazi N Siyal A Basit S Shammas M Al-Mukhaylid S Aleem A. Clinical validation of the somatic FANCD2 mutation (c.2022-5C>T) as a novel molecular biomarker for early disease progression in chronic myeloid leukemia: A case–control study. Hematol Rep. (2024) 16:465–78. doi: 10.3390/hematolrep16030045 PMC11270283 39051418

[130] Jain AG Nakitandwe J Desai K Crane GM Bosler DS O’Brien J. Impact of non-ABL1 mutations on outcomes in patients with chronic myeloid leukemia. Blood. (2024) 144:3153–3. doi: 10.1182/blood-2024-209165

[131] Schiffer CA. New patterns of genetic instability in chronic myeloid leukemia: interesting, but not ready for clinical use. Haematologica. (2023) 108:2273–4. doi: 10.3324/haematol.2023.283059 PMC10483338 37078263

[132] Branford S Hochhaus A Mauro M Minami Y Rea D Boquimpani De Moura Freitas CM. Impact of mutations in blood cancer-related genes on clinical outcomes in chronic myeloid leukemia in chronic phase (CML-CP) after ≥2 tyrosine kinase inhibitors (TKIs) in the ascembl trial. Blood. (2023) 142:449. doi: 10.1182/blood-2023-187636

[133] Soverini S De Santis S Monaldi C Mancini M Giai V Cerrano M. ASXL1 Mutations at Diagnosis Did Not Impact on the Depth of Molecular Response (MR) and on Treatment-Free Remission (TFR) Eligibility in Chronic Phase (CP) Chronic Myeloid Leukemia (CML) Patients (pts) Receiving either Nilotinib (NIL) First-Line or Imatinib (IM) with Early Switch to NIL in Case of No Optimal Response in the SUSTRENIM Clinical Trial. Blood. (2024) 144:3158–8. doi: 10.1182/blood-2024-205465

[134] Fernandes A Shanmuganathan N Branford S. Genomic mechanisms influencing outcome in chronic myeloid leukemia. Cancers. (2022) 14:620. doi: 10.3390/cancers14030620 35158889 PMC8833554

[135] Breccia M Efficace F Scalzulli E Ciotti G Maestrini G Colafigli G. Measuring prognosis in chronic myeloid leukemia: what’s new? Expert Rev Hematol. (2021) 14:577–85. doi: 10.1080/17474086.2021.1938534 34075852

[136] Branford S Fernandes A Shahrin NH Maqsood M Shanmuganathan N Wadham C. Beyond BCR::ABL1—The role of genomic analyses in the management of CML. J Natl Compr Cancer Netw. (2024) 22(1):e237335. doi: 10.6004/jnccn.2023.7335 38394774

[137] Zhang Y Bi H Wang Y Chen L Pan J Xu P. BCR-ABL1 is a secondary event after JAK2V617F in a patient with essential thrombocythemia who develop chronic myeloid leukemia. Blood Sci. (2022) 4:199. doi: 10.1097/BS9.0000000000000129 36518237 PMC9742103

[138] Kim TH Tyndel MS Kim HJ Ahn JS Choi SH Park HJ. Spectrum of somatic mutation dynamics in chronic myeloid leukemia following tyrosine kinase inhibitor therapy. Blood. (2017) 129:38–47. doi: 10.1182/blood-2016-04-708560 27733357

[139] Sobieralski P Bieniaszewska M Leszczyńska A Żuk M Wasąg B Zaucha JM. Secondary chronic myeloid leukemia in a patient with CALR and ASXL1-mutated primary myelofibrosis. Int J Hematol. (2022) 116:442–5. doi: 10.1007/s12185-022-03331-x 35429330

[140] Hu S Chen D Xu X Zhang L Wang S Jin K. Targeted next-generation sequencing identifies additional mutations other than BCR∷ABL in chronic myeloid leukemia patients: A chinese monocentric retrospective study. Cancers (Basel). (2022) 14:5752. doi: 10.3390/cancers14235752 36497234 PMC9739759

[141] Karantanos T Jain T Moliterno AR Jones RJ Dezern AE. Sex-related differences in chronic myeloid neoplasms: from the clinical observation to the underlying biology. Int J Mol Sci. (2021) 22:2595. doi: 10.3390/ijms22052595 33807519 PMC7961949

[142] Shanmuganathan N Wadham C Shahrin NH Feng J Thomson D Wang P. Impact of additional genetic abnormalities at diagnosis of chronic myeloid leukemia for first-line imatinib-treated patients receiving proactive treatment intervention. Haematologica. (2023) 108:2380. doi: 10.3324/haematol.2022.282184 36951160 PMC10483360

[143] Fei F Jariwala A Pullarkat S Loo E Liu Y Tizro P. Genomic landscape of myelodysplastic/myeloproliferative neoplasms: A multi-central study. Int J Mol Sci. (2024) 25(18):10214. doi: 10.3390/ijms251810214 39337700 PMC11431978

[144] Jafarbeik-Iravani N Kolahdozan S Esmaeili R. The role of ASXL1 mutations and ASXL1 CircRNAs in cancer. Biomarkers. (2024) 29:1–6. doi: 10.1080/1354750X.2024.2304187 38193494

[145] Guerineau H Cayuela J Dulucq S Tran Quang V Tarfi S Gricourt G. Mutation of epigenetic regulators at diagnosis is an independent predictor of tyrosine kinase inhibitor treatment failure in chronic myeloid leukemia: A report from the RESIDIAG study. Am J Hematol. (2024) 100(3):507–10. doi: 10.1002/ajh.27553 39654506

[146] Rinke J Chase A Cross NCP Hochhaus A Ernst T. EZH2 in myeloid Malignancies. Cells. (2020) 9:1639. doi: 10.3390/cells9071639 32650416 PMC7407223

[147] Bower H Björkholm M Dickman PW Höglund M Lambert PC Andersson TML. Life expectancy of patients with chronic myeloid leukemia approaches the life expectancy of the general population. J Clin Oncol. (2016) 34:2851–7. doi: 10.1200/JCO.2015.66.2866 27325849

[148] Carrà G Cartellà A Maffeo B Morotti A. Strategies for targeting chronic myeloid leukaemia stem cells. Blood Lymphat Cancer. (2019) 9:45. doi: 10.2147/BLCTT.S228815 31807112 PMC6842740

[149] Cavalleri A Xhahysa B Mutti S Ferraro RM Mazzoldi EL Farina M. Different *in vitro* models of chronic myeloid leukemia show different characteristics: biological replicates are not biologically equivalent. Cell Biol Int. (2025) 49(5):570–86. doi: 10.1002/cbin.70007 PMC11994880 40022557

[150] Adnan Awad S Dufva O Klievink J Karjalainen E Ianevski A Pietarinen P. Integrated drug profiling and CRISPR screening identify BCR::ABL1-independent vulnerabilities in chronic myeloid leukemia. Cell Rep Med. (2024) 5:101521. doi: 10.1016/j.xcrm.2024.101521 38653245 PMC11148568

